# The complete mitochondrial genome of *Nematobrycon palmeri* (Characiformes:Nematobrycon) and phylogenetic studies of Characidaes

**DOI:** 10.1080/23802359.2020.1825130

**Published:** 2020-10-05

**Authors:** Qi Wang, Tao Zhang, Xiaolong Yin, Fang Meng, Youkun Huang, Bingjian Liu, Yifan Liu

**Affiliations:** a National Engineering Research Center for Marine Aquaculture, Zhejiang Ocean University, Zhoushan, China; b National Engineering Laboratory of Marine Germplasm Resources Exploration and Utilization, Marine Science and Technology College, Zhejiang Ocean University, Zhoushan, China; c Zhejiang Province Key Lab of Mariculture and Enhancement, Marine Fisheries Research Institute of Zhejiang, Zhoushan, China; d Zhoushan Fisheries Research Institute of Zhejiang Province, Zhoushan, China

**Keywords:** *Nematobrycon palmeri*, mitochondrial genome, evolutionary relationships

## Abstract

Complete mitochondrial genome of the Characiform fish *Nematobrycon palmeri* was characterized in the present study. The whole mitogenome was 17,340 bp in size and the proportion of coding sequences with a total length of 11,448 bp was 66.02%, which encodes 3805 amino acids. The base composition of the genome was 30.92% for A, 23.92% for C, 14.88% for G, and 30.28% for T. All protein-coding genes were started with ATG, CO1 and ATP8 ended by AGG, TAG respectively, whereas CO2, ATP6, ND4 ended by a single T, the other PCGs commonly ended by TAA. The length of 12S and 16S ribosomal RNA was 949 bp and 1675 bp, respectively. The control region (D-loop) ranging from 15,654 bp to 17,340 bp was 1687 bp in size. It showed negative GC skew value (–0.2329) and positive AT skewness (0.0105). Phylogenetic analysis showed that *N. palmeri* was most closely related to *Gephyrocharax atracaudatus*. The complete mitochondrial genome sequence would provide a new insight into taxonomic classification, and help to draw a more complete picture of species diversity within the Characidae.

*Nematobrycon palmeri* belongs to the family Characidae and the order Characiformes, a species of characid fish found in the Atrato and San Juan river basins in western Colombia. Though diverse species based on anatomical diversity have been added to each genus, but there is hardly any sequences on the Nematobrycon. We are at loss here to assess properly the status of species of Nematobrycon relevant to our review because their descriptions are incomplete and/or misleading. In this study, we determined the complete mitochondrial genome of *N. palmeri*, which would supplement the limited data on molecular level and even be reference for systematics.

Specimens of *N. palmeri* were collected from San Juan river basin of Colombia (4°02′42″N, 77°26′29″W) and stored in a refrigerator of −80 °C in Zhejiang Engineering Research Center for Mariculture and Fishery Enhancement Museum (Accession number: PH181922). Total genomic DNA was extracted from muscle of three different individuals using the phenol–chloroform method (Barnett and Larson 2012). The calculation of base composition and phylogenetic construction were conducted by MEGA6.0 software (Tamura et al. 2013). The transfer RNA (tRNA) genes were generated with the program tRNAs-can-SE (Lowe and Eddy 1997). The mitochondrial genome sequence of *N. palmeri* was sequenced by Sanger dideoxy and assembled by CodonCode Aligner 5.1.5 (CodonCode Corporation, Dedham, MA), the annotated genes was deposited in GenBank with the accession number MN861079.

Similar to the typical mitogenome of vertebrates, the mitogenome of *N. palmeri* is a closed double-stranded circular molecule of 17,340 bp including 13 protein-coding genes, 2 ribosomal RNA genes, 22 tRNA genes and two main noncoding regions (Boore 1999; Zhu et al. 2018). The contents of A, C, G, and T are 30.92%, 23.92%, 14.88%, and 30.28%, respectively. Most mitochondrial genes are encoded on the H-strand except for ND6 and eight tRNA genes (Gln, Ala, Asn, Cys, Tyr, Ser, Glu, and Pro), which are encoded on the L-strand. The proportion of coding sequences with a total length of 11,448 bp is 66.02%, 13 protein-coding genes (PCGs) encode 3805 amino acids in total. A-T and G-C contents of mitochondrial genome were 61.21% and 38.79%, respectively, thereby with a high AT bias. Besides, it showed negative GC skew value (–0.2329), indicating that C base was more common than G base, whereas AT skewness was positive (0.0105), suggesting A base occurs more frequently than T base in the *N. palmeri* mitochondrial genome.

All the protein-coding genes use the initiation codon ATG, which is quite common in vertebrate mtDNA (Miya et al. 2001; Liu et al. 2017). ND1, ND2, CO3, ND3, ND4L, ND5, ND6 and CytB end by TAA as a stop codon, CO1 and ATP8 end by AGG, TAG respectively, and three incomplete termination codons (T) were found in the other genes (CO2, ATP6, and ND4). The content of Cys in 13 PCGs was the lowest accounting for 0.79% and there were 5 amino acids (from high to low: Leu 16.35%, Ala 8.36%, Thr 7.73%, Ile 7.28%, Phe 6.89%) used in a high frequency. In particular, the content of Leu was the highest.

The lengths of 12S ribosomal RNA and 16S ribosomal RNA were 949 bp and 1675 bp, which were both located in the typical positions between tRNA-Phe and tRNA-Leu (UUA), separated by tRNA-Val (Petrillo et al. 2006). The overall A + T content was 57.81%, the AT and GC skew value for the rRNAs were 0.2037 and −0.0894, respectively. The 22 *N. palmeri* mt-tRNA genes varied in size from 67 nucleotides to 75 nucleotides. Additionally, the length of control region (D-loop) was 1687 bp, ranging from 15,654 bp to 17,340 bp. The control region is also called A + T-rich region because of its high content of adenine and thymine, 620 nucleotides for A, 565 nucleotides for T, both of them were accounting for 70.24% of the whole D-loop, which was a usual large number higher than overall AT content (61.21%).

Neighbor-joining (NJ) tree of 15 Characidaes species was constructed based on 12 PCGs. The bootstrap values were based on 10,000 resamplings. The number at each node is the bootstrap probability. The result suggested that *N. palmeri* was most closely related to *Gephyrocharax atracaudatus* among all the Characidaes species included in the analysis. This result was consistent with conventional morphological taxonomy. In Figure 1, Characidaes were divided into three branches, Nematobrycon + Gephyrocharax was resolved as sister group of a clade formed by Oligosarcus + Astyanax + Paracheirodon + Grundulus in the combined data set within the tribe Characidae. The other genera were far more evolutionarily related than the above two clades. Our findings suggested that, the phylogenetic placement of *N. palmeri* was well supported within Characidae. We believe that more similar discoveries of these species would further promote better understand of the Characidae family.

**Figure 1.**
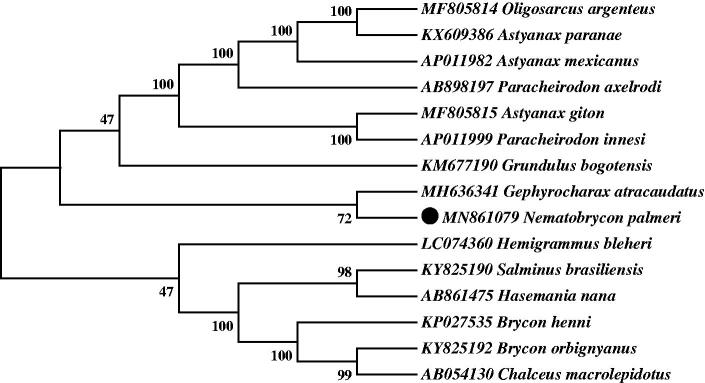
Neighbor-joining (NJ) tree of 15 Characidaes species based on 12 PCGs. The bootstrap values were based on 10,000 resamplings. The number at each node is the bootstrap probability. The number before the species name is the GenBank accession number. The genome sequence in this study is labeled with a black spot.

## Data Availability

The data that support the findings of this study are openly available at NCBI (https://www.ncbi.nlm.nih.gov), GenBank accession no. MN861079. And the data that support the findings of this study are also available from the corresponding author, Dr. Liu, upon reasonable request.
